# Multicenter assessment of impairments and disabilities associated with Beirut blast injuries: a retrospective review of hospital medical records

**DOI:** 10.1136/tsaco-2023-001103

**Published:** 2023-10-04

**Authors:** Samar Al-Hajj, Mahmoud El-Hussein, Johan von Schreeb, Cima Hamieh, Nesrine Ahmad, Nagi Souaiby

**Affiliations:** 1 Department of Epidemiology and Population Health, American University of Beirut, Beirut, Lebanon; 2 Lariboisière Hospital, Paris, France; 3 Department of Public Health Sciences, Division of Global Health (IHCAR), Karolinska Institute, Stockholm, Stockholm, Sweden; 4 Montfermeil Hospital, Montfermeil, France; 5 Middle East and North Africa Program for Advanced Injury Research, American University of Beirut, Beirut, Lebanon; 6 Saint Joseph University, Beirut, Lebanon

**Keywords:** epidemiology, Mass Casualty Incidents

## Abstract

**Objectives:**

This study aims to describe the injury patterns of the Beirut blast victims and assess hospitals’ disaster management and preparedness during the 2020 Beirut port blast.

**Methods:**

A cross-sectional retrospective multicenter study was conducted in two stages. Data were collected on blast victims presented to participating hospitals from August 4 till August 8, using three designed questionnaires. Stage 1 included all blast patients’ records and stage 2 examined a subset of inpatient and outpatient records. Binary logistic regression was performed to assess the factors associated with death and disability for blast patients.

**Results:**

A total of 3278 records were collected, 83% were treated at emergency departments and 17% were admitted to hospitals. Among those, 61 deaths and 35 long-term disabilities were reported. Extremity operations (63%) were mostly performed. Outpatients (n=410) had a mean age of 40±17.01 years and 40% sustained lacerations (40%). 10% of those patients sustained neurological complications and mental problems, and 8% had eye complications. Inpatients (n=282) had a mean age of 49±20.7 years and a mean length of hospital stay of 6±10.7 days. Secondary (37%) and tertiary (25%) blast injuries were predominant. 49% sustained extremity injuries and 19% head/face injuries. 11 inpatient deaths and 20 long-term disabilities were reported. Death was significantly associated with tertiary concussion and crush syndrome (p<0.05). Of the 16 hospitals, 13 implemented disaster plans (87%), and 14 performed a triage with a mean time of 0.96±0.67 hours. One hospital (6%) performed psychological evaluations, without follow-up.

**Conclusion:**

Beirut blast victims suffered deaths and disabilities associated with their injuries. They predominantly sustained lacerations caused by shattered glass. Tertiary injuries were associated with death. Triage, disaster plans, and hospital preparedness should be effectively implemented to enhance patients’ clinical outcomes.

**Level of evidence:**

Prognostic and epidemiological/Level III

**WHAT IS ALREADY KNOWN ON THIS TOPIC**

- The Beirut blast is the third largest non-nuclear explosion in modern times.
- This study provides in-depth understanding of injury patterns, clinical outcomes, and hospitals’ response plans for a surge of mass casualties in urban settings and occurring during the COVID-19 pandemic, and an unprecedented socioeconomic crisis.

**WHAT THIS STUDY ADDS**

- The findings indicated that most victims sustained mild injuries and were treated and discharged from the emergency department within the first 24 hours after the blast. Secondary laceration injuries were the most prevalent due to unlaminated shattered glass.
- This study revealed that death was associated with tertiary blast injuries.
- Most hospitals controlled the casualties, reflecting their high quality of healthcare, yet hospitals’ disaster response plans revealed gaps that should be addressed.

**HOW THIS STUDY MIGHT AFFECT RESEARCH, PRACTICE, OR POLICY**

- Synthesized knowledge from this study can help to guide efforts towards enhancing hospital preparedness and multidepartmental coordination during mass casualties.
- It further urges key policymakers and stakeholders to design and develop effective guidelines, regulations, and practices that support hospitals’ adequate and timely response to mass casualty incidents.

## Introduction

Mass casualty incidents (MCIs) are unpredicted public health disasters that impose significant burdens on the affected communities. The blast in Beirut on August 4, 2020, is one of the most devastating MCIs in modern history^1^ caused by the explosion of 2750 tons of inadequately stored ammonium nitrate (AN). The proximity of the blast epicenter to the capital’s densely populated area led to the deaths of more than 200 individuals, 6000 casualties, and massive destruction to more than 300 000 residential homes.^2 3^ The estimated economic cost of the blast exceeded 15 billion dollars in damages.^2^

Explosions, especially from high-order explosive materials like AN, impose acute injuries on the exposed population.^4^ Blast injuries caused by chemical explosive materials are classified into four categories: primary, secondary, tertiary, and quaternary.^4^ Primary and secondary blast injuries are induced by blast wave overpressure and flying debris, respectively.^4 5^ Tertiary blast injuries result from victims’ body being thrown away by the blast, whereas quaternary blast injuries constitute unclassified injuries, such as burns and intoxication.^4^

In addition to physical injuries, blasts result in significant psychological trauma, often manifested in severe anxiety, depression, and persistent post-traumatic stress disorder (PTSD) symptoms such as fear and altered response.^6^ Moreover, blast traumatic brain injury and concussion can cause direct or delayed chronic changes in the behavior and neurostructure of the diagnosed victim.^7^ After the Beirut blast, studies showed that an increasing number of people, including children, suffered from anxiety, PTSD, and/or depression.^8 9^

The Beirut explosion imposed a substantial burden on fragile local healthcare systems and drained their limited resources. MCIs often require adequate emergency preparedness and multidepartmental coordination to effectively handle the sudden surge in casualties and ultimately reduce fatalities.^3 10^ However, numerous challenges altered the effective management of the Beirut blast casualties. The fast-spreading COVID-19 pandemic, the absence of emergency medical services (EMS) in Lebanon, the protracted socioeconomic crisis, and the heterogenous destruction that affected six acute care hospitals and 22 healthcare facilities led to a substantial reduction in healthcare capacity and resources.^3 10–12^ The blast damaged buildings up to 10 km away from the blast epicenter, rendering three major urban hospitals unfunctional and reducing hospitals’ capacity by at least 500 beds.^11 13^ Hospital admitted patients were transferred from damaged hospitals and destroyed wards to peripheral hospitals by civilians and ambulances, exacerbating the pressure on the fragile infrastructure of local healthcare systems.^14^ Some patients were treated in hospital parking lots or under clinics’ rubbles using flashlights as a result of hospital emergency power failure.^15^ Despite their effects on the population and local healthcare systems, the epidemiology of blast injury and the hospital emergency responses during the blast remain understudied in Lebanon with limited impact on patient management during disasters and hospital preparedness and responses.

To address this gap, this study aims to assess and describe the Beirut blast victims’ injuries admitted to major acute care hospitals across Lebanon, mainly in terms of the injury’s clinical presentation, hospital procedures, and patients’ disability on discharge. It further evaluates the hospitals’ disaster and emergency response plans and management during the Beirut blast. Improving our understanding will help to inform the development of effective hospital healthcare systems policies and strategies for managing MCIs, particularly in low-income and middle-income countries with improper documentation and limited healthcare resources.

## Methods

### Study design

**Figure 1:**
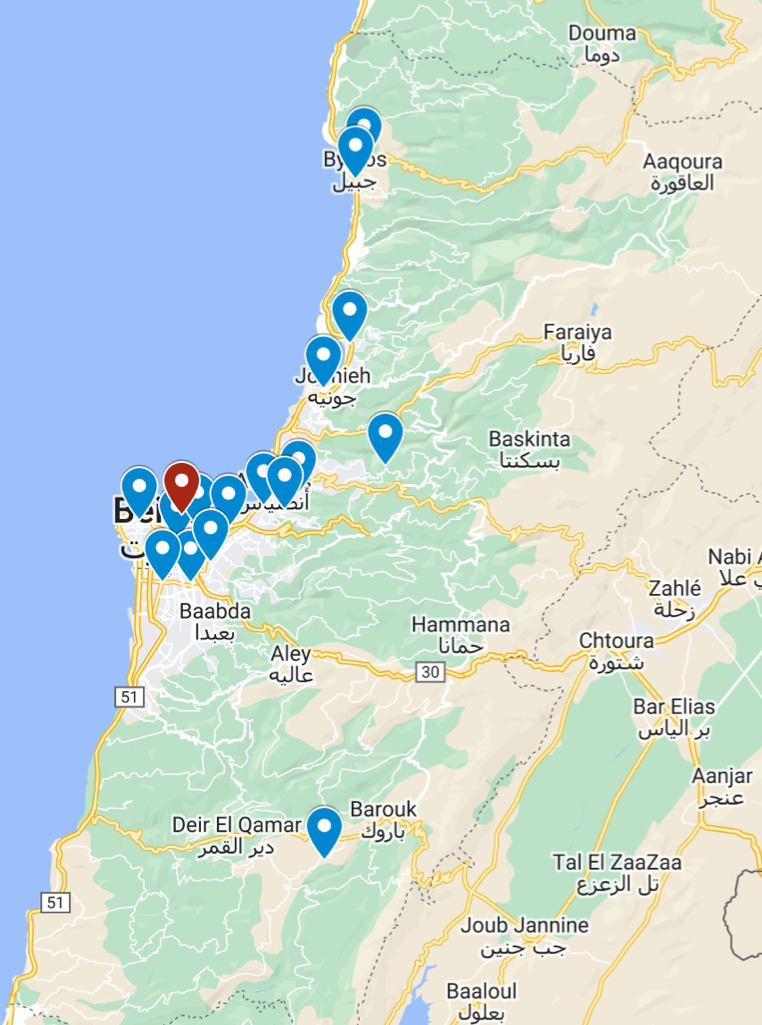
Map of all the participating hospitals (blue pin) with respect to the blast epicenter (red pin).

A multicenter, cross-sectional study was conducted in two stages across 16 geographically dispersed hospitals across Lebanon (figure 1): 4 hospitals located in Beirut, 4 hospitals in Mount Lebanon (≈10 km to 20 km away from the blast epicenter), and 8 hospitals in Metn (≈6 km away). Stage 1 assessed all blast patients’ records who were admitted to one of the participating hospitals with a blast-related casualty form the time of the blast (August 4, 2020, 18:07) until 96 hours after the blast (August 8, 2020). In stage 2, selected subsets of outpatients’ and inpatients’ records were analyzed. Due to the surge of injured victims and inadequate documentation, many treated patients left the hospitals unregistered. Accordingly, only registered outpatients with hospital records, compromising 15% of the total outpatient population, were included in stage 2. Likewise, only registered inpatients’ records with accurate, reliable, and high-quality injury data were included in stage 2, constituting 50% of the total inpatient population. Eligible patient records were identified by the participating hospitals and included in this study. This process guarantees the inclusion of robust and comprehensive data, enhancing the overall quality of the analysis. Patient hospital distribution records and hospital catchment areas were not analyzed due to incomplete hospital documentation, absence of governmental data, and limited EMS in Lebanon. The latter forced victims to drive and seek treatment in acute care hospitals distant from the blast epicenter, many of whom were included in this study.

### Data collection

Data were retrospectively collected from the participating hospitals using three developed and pilot-tested questionnaires (online supplemental appendix A). Questionnaires were built after regular meetings between NS and JvS, representing the WHO. All questionnaires were endorsed by the Lebanese Ministry of Public Health and were completed by a designated person (eg, administrative staff, physician, or nurse) at each hospital.

10.1136/tsaco-2023-001103.supp1 Supplementary data

In stage 1, hospital blast response and management information were collected using Questionnaire 1 along with detailed information on injured individuals. Questionnaire 1 focused on documenting the following variables: (1) The number of hospitals that received blast patients, (2) The number and types of major operations performed, (3) The number of traumatic amputations, (4) The number of severely disabled patients, (5) The number of COVID-19 screenings and positive patients, (6) Availability and implementation of hospital MCI response plan and triage, and (7) Psychological assessment and follow-up.

Data on inpatient and outpatient injury presentation and outcomes were collected using Questionnaires 2 and 3, respectively. Questionnaire 2 focused on identifying the following inpatient variables: (1) Patient’s demographics, (2) Hospital admission type, (3) Injury category, (4) Operation types, (5) COVID-19 status, (6) Intoxication status, (7) International Classification of Disease, Tenth Revision (ICD-10) codes, (8) Length of hospital stay (LOS), (9) Mortality rate, and (10) Functional disability at discharge. Questionnaire 3 focused on collecting the following outpatient variables: (1) Patient’s demographics, (2) Patients’ respiratory, ophthalmic, dermatologic, cardiac, neurologic, and/or psychologic complications.

Injury categories were identified according to the blast injury classifications defined by the Centers for Disease Control and Prevention (CDC). Blast injuries were categorized into primary, secondary, tertiary, and quaternary by the healthcare workers in the participating hospitals during assessment through history-taking and interrogation of the entourage whenever feasible.

### Patient and public involvement

No patient was involved.

### Data analysis

Data were analyzed via IBM Statistical Package for Social Sciences (V.29). Descriptive analyses were performed to assess physical blast injuries and the participating hospitals’ management and response plan. Findings were expressed in terms of count (N) and percent (%) for categorical variables and mean±SD for continuous variables. Binary logistic regression was performed to assess all factors associated with death and disability for inpatients’ data using a cut-off value of p≤0.05, 95% CI, and OR. Variables with less than 5% missing values were imputed by mean. Missing values (>5%) were not imputed and were excluded from the analysis. Missing CDC injury classification was considered unclassified.

## Results

A total of 3278 blast patients’ records were collected from 16 participating hospitals during stage 1, representing over 50% of the total blast-related casualties (n>6000).

Eighty-three percent were treated and discharged from the emergency departments (EDs; n=2714) and 17% were admitted to the hospital (n=564). Of the outpatients, 72.1% were treated and discharged within the first 24 hours after the blast (n=2365). Of the inpatients, 14.8% were admitted to regular hospital floors (n=486), and 2.4% required intensive care unit (ICU) admission (n=78). Sixty blast patients were declared dead, with most fatalities being reported on arrival (n=41; 1.3%). Thirty patients suffered from severe disability (0.9%), whereas five experienced traumatic amputations (0.2%). Operations commonly managed extremity injuries (n=190; 62.5%), neurological (n=64; 21.1%) injuries, and thorax/abdominal injuries (n=7; 2.3% each) (table 1).

**Table 1:** Distribution of injury characteristics of the general sample in phase I (n=3278)

Variable	N (%)
**Injury**	
Total number of injuries	3278 (100%)
**Patient disposition**	
ED treated and discharged	2714 (82.8%)
≤24 hours*	2365 (72.1%)
>24 hours*	349 (10.7%)
Hospital admission	564 (17.3%)
Floor	486 (14.8%)
ICU	78 (2.4%)
**Long-term disability**	
Severe disability	30 (0.9%)
Trauma amputation	5 (0.2%)
**Performed operations**	
Total number	304 (100%)
Major operations†	233 (76.6%)
≤24 hours*	159 (52.3%)
≤24 hours‡	74 (24.3%)
Type of operation§	
Neurooperation	64 (21.1%)
Thorax operation	7 (2.3%)
Abdominal operation	7 (2.3%)
Extremity operation	190 (62.5%)
Other	36 (11.8%)
**Death**	
Total number of deaths	60 (1.8%)
On arrival	41 (1.3%)
≤24 hours*	14 (0.4%)
>24 hours*	5 (0.2%)

*Time since the blast.

†Major operations (as defined within the questionnaire): an operation on an organ within the cranium, chest, abdomen, or pelvic cavity.

‡Time since the patient was admitted to the hospital.

§Type of operations (as defined in the questionnaire): neuro-operation (involving brain, spinal cord, ophthalmology, and ENT), thorax, abdomen (including the pelvis), and extremity (involving all the four limbs).

ED, emergency department; ICU, intensive care unit.

### Outpatient victims

A total of 410 outpatients were included in this study stage. The participants’ age ranged from 0 year to 92 years, with a mean age of 40±17.01 years. Of the cohort 42.2% are female and 56.1% male (table 2).

**Table 2:** Distribution of injury characteristics of the outpatient sample in phase II (n=410)

Variable	N (%)	Mean±SD
Outpatient sample size	410 (100%)*	
Gender		
Male	230 (56.1%)	
Female	173 (42.2%)	
Unknown	7 (1.7%)	
Age (years)		40±17.01
Type of injury†		
Concussion	22 (5.8%)	
Bruise	64 (16.9%)	
Cut	163 (43%)	
Sprain/strain/dislocation	11 (2.9%)	
Fracture	42 (11.1%)	
Wound	90 (23.8%)	
Other‡	50 (13.2%)	
Patients with multiple types	60 (15.8%)	
Respiratory complication		
Symptoms§	11 (2.7%)	
Other symptoms¶	5 (1.2%)	
Eye complication	32 (7.8%)	
Skin irritation	26 (6.3%)	
Cardiac complication	25 (6.1%)	
Neurological complication	42 (10.2%)	
Mental complication	41 (10%)	

*15% of the total outpatient sample.

†Patient can sustain multiple injuries.

‡Other include trauma, dyspnea, hematoma, foreign bodies, eye injury, tympanic membrane rupture, etc.

§Cough, wheeze, excessive phlegm production.

¶Other respiratory symptoms caused by high exposure to dust and/or air pollution.

Assessing the injury type sustained by the outpatients revealed that lacerations (43%), wounds (23.8%), and bruises (16.9%) were the most common. At least 16% of these patients reported multiple injury types. Of this cohort, 3.9% suffered from respiratory complications, 7.8% from ophthalmic, 6.3% from skin irritation, 6.1% from cardiac, 10.2% from neurological, and 10% from mental difficulties (table 2).

### Inpatient victims

A total of 564 inpatients were included in this study stage. Participants’ age ranged from less than 1 year to 93 years, with a mean age of 49±20.7 years. Of these patients 52.8% were male (n=149) and 47.2% were female (n=133). Most victims were admitted to regular floors (84.8%) and only 15.2% required ICU, with a mean LOS of 6±10.7 days. Out of the 11 reported deaths (3.6%), 6 inpatients died as a result of their blast injury (2.1%) and 5 patients died postoperatively due to organ failure (1.8%). Of this cohort, 12.2% suffered from a disability on discharge. Extremity injuries (49.3%), and head/face injuries (19.3%) were frequently diagnosed among inpatient victims (table 3; online supplemental appendix B, table B1).

10.1136/tsaco-2023-001103.supp2 Supplementary data

**Table 3:** Summary of the distribution of injury characteristics of the inpatient sample in phase II (n=282) (full length table can be found in online supplemental appendix B, table B1)

Variable	N (%)	Mean±SD
Inpatient sample size	282 (100%)	
Gender		
Male	149 (52.8%)	
Female	133 (47.2%)	
Age (years)		49±20.7
Injured body part		
Extremities	69 (49.3%)	
Abdomen/thorax	3 (2.1%)	
Head/face	27 (19.3%)	
Spine	5 (3.6%)	
Multiple regions	28 (20%)	
Hospital admission		
Floor	139 (84.8%)	
ICU	25 (15.2%)	
Injury category		
Primary	40 (14.2%)	
Concussion	36 (12.8%)	
Blast lung	3 (1.1%)	
Tympanic membrane rupture	1 (0.35%)	
Secondary	105 (37.2%)	
Lacerations	49 (17.4%)	
Concussion	16 (5.7%)	
Penetrating injury (including eye injuries)	30 (10.6%)	
Traumatic amputation	8 (2.8%)	
Tertiary	69 (24.47%)	
Blunt injury	45 (16%)	
Concussion	19 (6.7%)	
Crush syndrome	5 (1.8%)	
Quaternary	22 (7.8%)	
Burns	4 (1.4%)	
Toxic gas inhalation	2 (0.71%)	
Environmental contamination	16 (5.7%)	
Operation and minor procedures	165 (58.5%)	
Length of hospital stay (days)		6±10.7
Death		
Immediate/blast-related	6 (2.1%)	
Postoperative procedure (organ failure)	5 (1.8%)	
Long-term disability at discharge	20 (12.2%)	
ICD-10 code occurrence		
S00-S09 (injuries to the head)	19 (13.6%)	
S50-S59 (injuries to the elbow and forearm)	21 (15%)	
S60-S69 (injuries to the wrist)	20 (14.3%)	
T00-T07 (injuries involving multiple body regions)	18 (12.9%)	

ICD-10, International Classification of Disease, Tenth Revision; ICU, intensive care unit.

Most patients had secondary blast injuries (n=105, 37.2%), particularly lacerations (17.4%), penetrating injuries (10.6%), and concussions (5.7%). Of the patients 24.5% sustained tertiary blast injuries (n=69), common blunt injuries (16%), and concussions (6.7%). Primary blast injuries were common (n=40; 14.2%), particularly concussions (12.8%). Only 7.8% of the patients’ injuries were quaternary (n=22), mainly due to environmental contamination (5.7%) and burns (1.4%) (table 3; online supplemental appendix B, table B1).

Injury ICD-10 codes were the primarily documented, particularly injury to the elbow and forearm (S50–S59; 15%), wrist and hand (S60–S69; 14.3%), head (S00–S09; 13.6%), and multiple body parts (T00–T07; 12.9%) (table 3; online supplemental appendix B, table B1).

The logistic regression model revealed that death was significantly associated with tertiary concussions (p=0.047) and tertiary crush syndrome (p=0.021). Injuries associated with increased odds of death included ‘sustaining multiple injury categories’ (OR=1.707, 95% CI 0.421 to 6.915), ‘primary blast lung injuries’ (OR=3.714, 95% CI 0.205 to 67.149), ‘tertiary blunt injuries’ (OR=1.297, 95% CI 0.236 to 7.132), ‘tertiary crush syndrome’ (OR=24, 95% CI 1.615 to 356.635), ‘quaternary burns’ (OR=6.6, 95% CI 5.543 to 80.235), and ‘injuries to the extremities’ (OR=1.855, 95% CI 0.208 to 16.501) and ‘spine’ (OR=11.333, 95% CI0. 765 to 167.501). Sustaining multiple injury categories (OR=1.891, 95% CI 0.473 to 7.564), secondary concussions (OR=2.625, 95% CI 0.22 to 31.349), secondary penetrating injuries (OR=1.667, 95% CI 0.257 to 10.792), tertiary crush syndrome (OR=1.167, 95% CI 0.094 to 14.518), and spine injuries (OR=2.167, 95% CI 0.262 to 17.892) increased the likelihood of patients sustaining a disability, though the association was insignificant (p>0.05) (table 4; online supplemental appendix B, table B2).

**Table 4:** Summary of the binary logistic regression model results predicting death and disability among inpatients (full length table can be found in online supplemental appendix B, table B2)

Variable	Unadjusted OR	95% CI	P value
**Death**			
**Injury category**			
Primary	0.795	0.39 to 1.619	0.527
Concussion	–	–	0.674
Blast lung	3.714	0.205 to 67.149	0.374
Secondary	1.335	0.866 to 2.060	0.191
Traumatic amputation	–	–	0.998
Penetrating injury	0.417	0.074 to 2.371	0.323
Tertiary	1.345	0.856 to 2.114	0.199
Concussion	–	–	0.047*
Blunt injury	1.297	0.236 to 7.132	0.765
Crush syndrome	24.000	1.615 to 356.635	0.021*
Quaternary	1.85	0.45 to 7.603	0.394
Environmental contamination	–	–	0.334
Burns	6.600	5.543 to 80.235	0.139
**Injured body part**			
Multiple	–	–	0.454
Extremities	1.855	0.208 to 16.501	0.58
Spine	11.333	0.765 to 167.971	0.078
**Disability**			
**Injury category**			
Primary	1.874	0.531 to 6.612	0.329
Concussion	–	–	1
Blast lung	0	0	1
Secondary	1.146	0.440 to 2.981	0.78
Traumatic amputation	–	–	0.652
Concussion	2.625	0.22 to 31.349	0.446
Lacerations	0.946	0.095 to 9.378	0.962
Penetrating injury	1.667	0.257 to 10.792	0.592
Tertiary	0.812	0.331 to 1.991	0.649
Concussion	–	–	0.455
Blunt injury	0.412	0.09 to 1.881	0.252
Crush syndrome	1.167	0.094 to 14.518	0.905
Quaternary	0	0	0.999
Environmental contamination	–	–	1
Burns	0	0	0.999
**Injured body part**			
Multiple	–	–	0.301
Head/face	0.464	0.072 to 2.976	0.418
Extremities	0.236	0.052 to 1.072	0.062
Spine	2.167	0.262 to 17.892	0.473

*Significance (p≤0.05).

### Hospital management and preparedness

Most hospitals managed to implement and verify COVID-19 screening tests for the presented patients (n=11; 68.8%). The verified PCR results revealed three positive patients only. Although 93.8% of the hospitals confirmed the availability of an MCI response plan (n=15), only 86.7% of these hospitals implemented their plan during the Beirut blast (n=13). Triage was reportedly executed by 87.5% of the participating hospitals (n=14) and was performed by physicians at two of the reporting triage-positive hospitals (14.3%) and by both physicians and nurses at 12 hospitals (85.7%) with a mean time of 0.96±0.67 hours to starting the triaging. Only one hospital documented psychological evaluation of the victims (6.3%), though without any further follow-up (n=0, 0%) (table 5).

**Table 5:** Details on the hospitals’ management and preparedness (n=16)

Variable	N (%)	Mean±SD
Total number of hospitals	16 (100%)	
PCR		
Yes	11 (68.8%)	
*Positive patients*	*3 patients*	
No	5 (31.3%)	
Mass casualty plan		
Yes	15 (93.8%)	
Implemented plan	13 (86.7%)	
No	1 (6.3%)	
Triage		
Yes	14 (87.5%)	
Time to start triage (hours)		0.96±0.67
Triage personnel		
Doctor	2 (14.3%)	
Nurse	0 (0%)	
Both	12 (85.7%)	
No/not documented	2 (12.5%)	
Triage personnel		
Doctor	2 (12.5%)	
Nurse	0 (0%)	
Both	12 (75%)	
Psychological evaluation		
Yes	1 (6.2%)	
No/not documented	15 (93.8%)	
Psychological follow-up		
Yes	0 (0%)	
No/not documented	16 (100%)	

## Discussion

This study describes Beirut blast patients’ injury characteristics and clinical outcomes across 16 major hospitals in Lebanon. It further assessed hospitals’ preparedness and response plans during an MCI. Synthesized findings from this study are critical to understanding AN-related injury characteristics and patterns, particularly in urban settings, with the aim to inform protocols for hospitals to improve their response strategies, especially in low-income and middle-income countries.

Throughout history, more than 30 AN-related explosions occurred globally, with varying strengths and outcomes, namely in Oppau (Germany, 1921), Texas (USA, 1947), and Tianjin (China, 2015).^3 16^ Contrary to the Beirut blast epicenter, the majority of these blasts happened in industrial settings and transportation sites.^3^ The central location of Beirut port and its proximity to the residential neighborhood resulted in the elevated number of casualties, with over 50% of the victims included in the first stage.

Consistent with the injury patterns reported in other open-air and urban MCIs, non-critical injuries were prevalent among Beirut blast victims.^17 18^ Collectively, the blast location, surge, and hospital management explain the relatively high rate of outpatients and immediate deaths. Yet, several studies suggested that the explosion’s timing amidst COVID-19 restrictions and after working hours (ie, 18:08), and the open-air setting limited the damages. Moreover, the massive grain silos bordering the stored AN, and the widespread concrete structures and residential homes across the Beirut metropolis absorbed the propagation of the blast shock wave and limited its impact on casualties’ severity.^19 20^

Mildly injured victims were able to reach the hospitals first and unassisted (spontaneous evacuations), explaining outpatients’ immediate surge. One study suggested that the first wave of mild casualties delayed the care for later-arriving severe injuries, plausibly explaining the high toll of death on arrival and within the first 24 hours.^3^ Nevertheless, as in other MCIs like 9/11 and the Oklahoma City bombing, most deaths occurred near ground zero and thus were excluded due to delayed extrication.^18 21^ The gradual drop in the death rate and the ≈3% postoperative death rate, which is comparable to USA’s ratio (0.57% to 2.1%), corroborates the proficiency of the medical staff.^22^ Besides the sparse critical patients, the latter justifies the relatively low hospital LOS compared with that of the Madrid bombings.^18^

Consistent with existing literature, secondary injuries are the leading cause of injuries in the Beirut blast^17 21 23 24^ due to the open-air blast nature, where secondary injuries constituted 84% of the Tianjin explosion’s injuries. Urban explosions, like the Oklahoma City bombing and the Beirut blast, notably reported secondary injuries due to propelled shrapnel fragments from windows and damaged building structures, particularly from unlaminated glass, causing lacerations and penetrating injuries.^3 25^ As reported in the current study, secondary injury settings commonly impacted the head, face, and extremities, and occasionally required hospitalization and operations, notably neuro-operations and extremity operations.^3 24^ The chaos and attention-grabbing initial explosion at the Beirut port few minutes before the massive blast, ignited individuals’ curiosity to move closer to residential windows and balconies instead of seeking protection before the onset of the second massive explosion, worsening victims’ injuries.^26^ In addition, victims’ exposure contributed to a number of secondary concussions as a result of flying objects from the overpressurized wave.^27^ Although the CDC claims that secondary blast injuries are the primary cause of death during a blast, this study did not show such an association.^28^ A plausible explanation is that the medical staff might have overlooked the process of identifying and documenting secondary blast injuries of patients suffering from additional severe injuries, such as blast lungs and fractures.

Similar to other AN explosions like in Tianjin and West Texas, this study revealed that tertiary blast injuries were common among Beirut blast victims.^11 17^ The blast wind, especially in open-air crowded residential areas, inflicts serious polytrauma on the victim’s body.^17 21^ The structural collapse that occurred near the blast epicenter is further linked to tertiary blast injuries, particularly crush injuries and concussions.^5 29^ A recent study reported that almost half of the patients sustaining crush injuries and reaching the hospital alive suffered from crush syndrome, a common cause of delayed post-injury mortality with the absence of immediate detection and treatment.^30^ This justifies the association between death and crush syndrome, as immediate detection and multidepartmental intervention were unlikely.

Primary blast injuries are thought to be underreported as the blast overpressure mostly affects victims near ground zero.^24^ This explains the insignificant association between primary blast injuries and mortality as the CDC confirms that primary blast injuries, particularly blast lung, increase the risk of death.^31^ The absence of classification of outpatient injuries, the incomplete inpatient data, and the delayed onset of many symptoms (ie, intoxication), hindered the classification of all injuries, particularly quaternary blast injuries.

Similar to earlier AN blasts, this study showed that the inpatient deaths and disabilities were affected by the collective blast forces, inflicting polytrauma on multiple body regions.^3 16^ Although this study revealed that spine injuries are associated with death and disability, proper short-term and long-term treatment helped reverse spine injury-induced disabilities, mostly attained in younger patients.^32–34^

### Hospital management and preparedness

Several urban hospitals were partially or fully destroyed, limiting their functionality and capacity for care provision.^3 17^ The layered burdens of treating blast victims, non-blast patients, and COVID-19 patients restricted many hospitals.^3^ Notably, various protocols, including psychological evaluation, follow-ups, formal triaging tools, and precaution practices, like COVID-19 screening and personal protective equipment, were mostly disregarded.^11 24^

Despite the implementation of emergency response plans by most hospitals, one study claimed that hospitals were unprepared for this overwhelming surge.^11^ Although triage helped alleviate these challenges, this study showed that the time taken to start the triage was greater than the ideal time.^35^ This may have increased death on arrival and in the ED. The surge and delayed triage further hindered the documentation and tracking of the patients’ records and thus the emergency care.^3^ Although most hospitals successfully performed COVID-19 screening to control the virus spread, this may have delayed and reduced staff performance. Even with the chaos, challenges, and limited resources, this study confirms that hospitals successfully controlled the mortality and disability rates.

Given the long-term mental and physical impacts of traumatic events, this study proved that hospitals failed to address the victims’ short-term and long-term mental effects. The mental impact of MCIs affects individuals who are physically injured as well as the witnesses, hence psychological assistance was deemed critical after the Madrid bombings.^17 18^ Accordingly, behavior change, anxiety, and PTSD, among other psychological illnesses, should be followed up and treated.

The study has some strengths and limitations. The nature of this multicenter study, which included 16 major acute care hospitals, enabled the establishment of a unique injury database and registry for the Beirut blast. The synthesized knowledge from this massive manmade explosion in urban sites is critical to emphasize the importance of safe chemical storage away from residential areas and the importance of educating people on their individual and social responsibilities during MCI. This is necessary especially in low- and middle- income countries (LMICs) with limited resources and access to EMS. By identifying the gaps in the hospital emergency plans, hospitals can enhance their disaster and emergency preparedness and responses, by training their staff, adopting more efficient triage systems, increasing their resources and improving their post-blast interventions. The most important post-blast interventions include long-term mental healthcare, rehabilitating and monitoring disabled victims, programming ambulatory wound care, and screening for wound infection. Social and economic support is also crucial to help the affected individuals return to life preblast.

This study’s retrospective nature limited the outcomes. Missing data due to the lack of proper documentation hindered this study. The sudden surge of casualties and hospitals’ destruction prevented patient reliable registration and proper documentation, which further affected the representation of the collected data. Additionally, due to the emergency nature of the blast, medical staff might have misreported many of the patients’ injuries, particularly the outpatient records. The absence of standardized disaster alert notices adopted by hospitals, as well as electronic documentation in some hospitals in Lebanon, further created a discrepancy in the data collection. Moreover, despite training the data collectors and following up with them, many centers had inconsistencies and incomplete data. Another limitation is related to the diagnosis and follow-up on mental illnesses, especially that many victims may have experienced a delayed onset of PTSD symptoms. Victims might also experience delayed onset of tympanic membrane rupture symptoms, explaining why only one patient was reported in our study. The lack of follow-up on physical injuries and disabilities further limited this study. Underreporting injuries, particularly primary blast injuries due to delayed extrication near the epicenter and not performing autopsies on the deceased, represents an additional limitation that might have affected the outcome of this study.

## Conclusion

This study presented the characteristics and long-term clinical outcomes of Beirut blast victims. This large-scale disaster drained the limited resource healthcare system and exposed its vulnerability in emergency preparedness and disaster planning. Findings from this study call for evaluating the public and private-sector disaster preparedness plans and for assisting hospitals in implementing efficient emergency responses during large-scale disasters. Necessary changes should be initiated, including prioritizing the training of medical personnel, both in hospital settings and in the field. A key area of focus should be triage training to ensure effective prioritization and management of patients. Additionally, attention should be given to less apparent injuries, such as tympanic membrane rupture and poisoning, to ensure their detection and appropriate treatment. In addition to addressing physical injuries, it is essential to provide psychological healthcare assessment and follow-up for patients at high risk. This includes patients who sought emergency room care, individuals who experienced shock within their homes, as well as the medical and evacuation staff who were involved in the response efforts. By implementing these measures, we can enhance the overall preparedness and response to such incidents, ensuring comprehensive care for both physical and psychological well-being. The study urges taking action and following up on blast victims and healthcare workers alike, particularly those who suffered post-blast anxiety and stress. This is crucial to understanding the long-term implications of MCIs on individuals, the healthcare system, and the society.

## Data Availability

Data are available upon reasonable request.
