# Supplementary material for: Multicenter assessment of impairments and disabilities associated with Beirut blast injuries: a retrospective review of hospital medical records
Source: Trauma Surg Acute Care Open. 2023 Oct 4;8(1):e001103. doi: 10.1136/tsaco-2023-001103 (PMC10551996; doi:10.1136/tsaco-2023-001103)
Supplement: Supplementary data [file tsaco-2023-001103supp001.pdf]

Supplementary Material-Appendix A

Questionnaire 1:

Hospital related questions
الأسئلة المتعلقة بالمستشفى

1. Please provide the name of the institution you work in:
يرجى تحديد اسم المؤسسة التي تعمل بها

2. Select your position in the hospital: (Admnistrative, Medical Doctor or a Registered Nurse)
حدد وظيفتك في المستشفى: (إداري/ة, طبيب/ة, ممرض/ة مسجل/ة)

PLEASE PROVIDE AN ANSWER OR N/A IF ANSWER NOT AVAILABLE
يرجى تقديم إجابة أو لا ينطبق إذا لم يكن الجواب متاحًا

Questions related to overall data of the explosion:
أسئلة تتعلق بالبيانات الإجمالية ليلة الانفجار

`

**number of patients divided (In-patients and out-patients)**<br>**(عدد المرضى مقسم إلى (الداخليون و الخارجيون)**	**Within first 24 hours of the explosion**<br>**خلال الـ 24 ساعة الأولى من الانفجار**	**After 24 hours of the explosion**<br>**بعد 24 ساعة من الانفجار**
1. How many patients were treated in the emergency department for minor injuries, and discharged afterwards?<br>كم عدد المرضى الذين عولجوا في قسم الطوارئ لإصابات طفيفة وخرجوا من المستشفى بعد ذلك؟		
2. How many patients were admitted to regular floor?<br>كم عدد المرضى الذين نقلوا الى الطوابق؟		
3. How many patients were admitted to the Intensive Care Unit?<br>كم عدد المرضى الذين تم قبولهم في وحدة العناية المركزة؟		
**Total Number of Admissions to the hospital (regular floor + ICU)**<br>**(إجمالي عدد حالات الدخول إلى المستشفى (طابق عادي + وحدة العناية المركزة**	**0**	
**How many injured patients did you receive in total ?**<br>**كم عدد المصابين الذين استقبلهم المستشفى إجمالاً ليلة الانفجار؟**	**0**	

**4. Major Surgeries***<br>**العمليات الجراحية الكبرى**	**Within first 24 hours**<br>**خلال الـ 24 ساعة الأولى**	**After 24 hours of admission**<br>**بعد 24 ساعة من الدخول**

**Total Number of Major Surgeries**<br>**إجمالي عدد العمليات الجراحية الكبرى**	**0**

**5. Type of Surgery****<br>**نوع العملية الجراحية**	**Neuro/جراحة الاعصاب**	**Thorax/الصدر**	**Abdominal/في البطن**	**Extremity/ جراحة الأطراف**
**Total Number of Surgeries**<br>**إجمالي عدد العمليات الجراحية**	**0**			

6. Was COVID-19 PCR done on the admitted patients to regular floor and ICU? Yes or No question<br>هل تم إجراء COVID-19 PCR للمرضى الذين تم إدخالهم إلى الطابق العادي ووحدة العناية المركزة؟ الإجابة بنعم أو لا<br>7. Were results verified? Yes or No question<br>هل تم التحقق من النتائج؟ الإجابة بنعم أو لا<br>8. If PCR was done, what is the number of COVID-19 Positive admited patients?<br>إذا تم إجراء PCR، فما هو عدد المرضى الذين اتت نتائجهم إيجابية وتم إدخالهم إلى المستشفى	

9. How many patients had a Major Amputation?***<br>عدد المرضى الذين خضعوا لعملية بتر كبرى	

10. How many patients were Severely Disabled?****<br>عدد المرضى الذين أصيبوا بإعاقة جسدية شديدة بسبب الانفجار	
11. Do you have a mass casualty plan? Yes or No question<br>هل لديك خطة لحوادث الإصابات الجماعية؟الإجابة بنعم أو لا<br>12. If Yes, was it implemented?<br>إذا كانت الإجابة بنعم ، فهل تم تنفيذها؟<br>13. Due to the rapid influx and high number of patients, how long did it take you to start triage? (in hours)<br>نظرًا لسرعة تدفق المرضى وعددهم الكبير، ما المدة التي استغرقها البدء بالفرز؟ (بعدد الساعات)	
14.Who did the triage? A nurse? A medical doctor? or both ?<br>من قام بالفرز؟؟ ممرض/ة؟ طبيب/ة؟ او كلاهما ؟	
15. Were there psychiatric evaluation for the admitted patients? Yes or No question<br>هل تمّ القيام بتقييم نفسي للمرضى المقبولين؟الإجابة بنعم أو لا<br>16. Were there psychiatric follow up for the discharged patients? Yes or No question<br>هل كانت هناك متابعة نفسية للمرضى الذين خرجوا من المستشفى؟الإجابة بنعم أو لا	
17. How many patients were dead upon arrival to the Emergency Department?<br>عدد المرضى الذين توفوا عند وصولهم لقسم الطوارئ	

18. How many patients died in the Emergency Department?<br>عدد المرضى الذين ماتوا في قسم الطوارئ	
19. How many patients died within 24 hours of admission?<br>عدد المرضى الذين ماتوا خلال 24 ساعة من الدخول	
20. How many patients died after 24 hours of admission?<br>عدد المرضى الذين ماتوا بعد 24 ساعة من الدخول	
**Total Number of Deaths/العدد الإجمالي للوفيات**	**0**

*Major Surgeries: an operation upon an organ within the cranium, chest, abdomen, or pelvic cavity, / العمليات الكبرى: عملية داخل الجمجمة أو الصدر أو البطن أو تجويف الحوض
**Type of surgeries: Neuro (involving brain, spinal cord, Ophtalmo and ENT), Thorax, Abdomen (including Pelvis) and Extremity (involving all the 4 limbs).
نوع العمليات الجراحية: عصبية (تشمل الدماغ والحبل الشوكي وجراحة العيون والأنف والحنجرة) , الصدر ,البطن (بما في ذلك الحوض) والأطراف (تشمل الأطراف الأربعة)
***Major Amputation: to cut (a part, such as a limb) from the body, Hand and above, Foot and above./ عملية البتر الكبرى: عملية قطع (جزء ، مثل الطرف) من الجسم ، من اليد وما فوق ، القدم وما فوق
****Severely Disabled: impaired or limited by a physical, mental, cognitive, or developmental condition. / لإعاقة الشديدة: أُضعِف او قُيِّد بسبب حالة بدنية أو عقلية أو إدراكية أو تنموية

Questionnaire 2:

Phase II. Individualized Questionnaire for Out-Patients	
Insitution:	
Patient's ID:	
Questions related to Public Health	***Answers***
1. Any patients who specifically developed cough, wheeze and excessive phlegm production<br>***2. Other respiratory symptoms caused by exposure to dust / higher levels of air pollution***<br>***3. Eye problems (irritation, erythema, secretions…)***<br>***4. Skin irritations / reaction (including delayed reaction, rash,)***<br>***5. Any cardiac events (e.g. chest pain, palpitation, dyspnea...)***<br>***6. Any atypical neurological effects (somatisation, headaches, tinnitis, imbalance, tinnitis…)***	
Questions related to Mental Health	***Answers***
1. Any record of significant mental health / psychological distress (anxiety, depression, insomina, paranoia...)	

Questionnaire 3:

**Phase II. Individualized Questionnaire for In-patients**	
**Insitution:**	
**Patient's ID:**	
**Questions related to Public Health**	**Answers**
1. Admission to Regular Floor or ICU<br>2. Were the patient operated?<br>3. Injury category (Primary, Secondary, Tertiary, Quaternary)*:<br>4. COVID status (positive or negative):<br>5. Toxic inhilation symptoms:<br>6. Length of stay in the hospital:<br>7. Diagnosis:<br>8. ICD 10 Code:<br>9. Death in Hospital:<br>10. Disability after discharge: IF yes, What is it?	
In case they want a **free** evaluation/re-evaluation	

*1.Primary: Tympanic membrane rupture, Blast Lung, Eye Injury, Concussion
2. Secondary: Penetrating Injury, Traumatic amputations, Lacerations, Concussion, iatrogenic
3. Tertiary: Blunt Injuries, Crush Syndrome, Compartment Syndrome, Concussion
4. Quaternary: Burns, Toxic gas or other inhalation injury, Injury-env. contamination
