# Supplementary material for: Multicenter assessment of impairments and disabilities associated with Beirut blast injuries: a retrospective review of hospital medical records
Source: Trauma Surg Acute Care Open. 2023 Oct 4;8(1):e001103. doi: 10.1136/tsaco-2023-001103 (PMC10551996; doi:10.1136/tsaco-2023-001103)
Supplement: Supplementary data [file tsaco-2023-001103supp002.pdf]

Supplementary Material- Appendix B

Table B1 Distribution of injury characteristics of the in-patient sample in phase 2 (N=282).

Variable	N (%)	Mean ± SD
**In-patient Sample Size**	282 (100%)*	
**Gender**		
Male	149 (52.8%)	
Female	133 (47.2%)	
**Age (Years)**		49± 20.7
**Injured Body Part**		
Extremities	69 (49.3%)	
Abdomen/Thorax	3 (2.1%)	
Head/Face	27 (19.3%)	
Spine	5 (3.6%)	
Multiple Regions	28 (20%)	
Other Regions	8 (5.7%)	
**Hospital Admission**		

Floor	139 (84.8%)	
ICU	25 (15.2%)	
**Injury Category** †		
Primary	40 (14.2%)	
Concussion	36 (12.8%)	
Blast Lung	3 (1.1%)	
Tympanic Membrane Rupture	1 (0.35%)	
Secondary	105 (37.2%)	
Lacerations	49 (17.4%)	
Concussion	16 (5.7%)	
Penetrating Injury (including eye injuries)	30 (10.6%)	
Traumatic Amputation	8 (2.8%)	
Pneumothorax	2 (0.71%)	
Tertiary	69 (24.47%)	
Blunt Injury	45 (16%)	
Concussion	19 (6.7%)	

Crush Syndrome	5 (1.8%)	
Quaternary	22 (7.8%)	
Burns	4 (1.4%)	
Toxic Gas Inhalation	2 (0.71%)	
Environmental Contamination	16 (5.7%)	
Number of Injury Category Classes		
Other/Not Documented	166 (58.9%)	
1 Category	51 (18.1%)	
≥ 2 Categories	65 (23%)	
**Surgery and Minor Procedures**	165 (58.5%)	
**Length of Hospital Stay (Days)**		6±10.7
**Death**		
Total in-patient deaths (Floor and ICU)	11 (3.6%)	
Immediate/ blast-related	6 (2.1%)	
Post-operative procedure (organ failure)	5 (1.8%)	
**Long-Term Disability at Discharge**	20 (12.2%)	

**ICD-10 Code**		
Number of patients with reported ICD-10	140 (49.6%)	
Code Occurrence ‡		
S00-S09 (injuries to the head)	19 (13.6%)	
S10-S19 (injuries to the neck)	6 (4.3%)	
S20-S29 (injuries to the thorax)	3 (2.1%)	
S30-S39 (injuries to the abdomen, lower back, lumber spine, pelvis, and genitals)	5 (3.6%)	
S40-S49 (injuries to the shoulder and upper arm)	12 (8.6%)	
S50-S59 (injuries to the elbow and forearm)	21 (15%)	
S60-S69 (injuries to the wrist)	20 (14.3%)	
S70-S79 (injuries to the hip and thigh)	8 (5.7%)	
S80-S89 (injuries to the knee and lower leg)	7 (5%)	
S90-S99 (injuries to the ankle and foot)	5 (3.6%)	
T00-T07 (injuries involving multiple body regions)	18 (12.9%)	
H15-H22 (disorders of the sclera, cornea, iris, and ciliary body)	5 (3.6%)	
W20-W49 (exposure to inanimate mechanical forces)	7 (5%)	

M60-M79 (soft tissue disorders)	3 (2.1%)	
Other	14 (10%)	

* 282 out of 564 total inpatient data (50%).

† Patients sustaining multiple injuries can be classified into more than one category.

‡ Number of patients classified with a certain ICD-10 code from the total in-patient sample size (N=282). A patient's multiple diseases and injuries can be classified by multiple relevant ICD-10 codes.

Table B2 Binary logistic regression model results predicting death and disability among in-patients.

Variable	Unadjusted OR †	95% CI †	p-value
**Death**			
**Injury Category**			
Primary	0.795	0.39-1.619	0.527
Concussion	--	--	0.674
Blast Lung	3.714	0.205-67.149	0.374
Tympanic Membrane Rupture	0	0	1
Secondary	1.335	0.866-2.060	0.191
Traumatic Amputation	--	--	0.998
Concussion	0	0	0.999
Lacerations	0	0	0.997
Penetrating Injury	0.417	0.074-2.371	0.323
Tertiary	1.345	0.856-2.114	0.199
Concussion	--	--	0.047*
Blunt Injury	1.297	0.236-7.132	0.765

Crush Syndrome	24.000	1.615-356.635	0.021*
Quaternary	1.85	0.45-7.603	0.394
Environmental Contamination	--	--	0.334
Burns	6.600	5.543-80.235	0.139
Toxic Gas Inhalation	0	0	1
Multiple Categories	1.707	0.421-6.915	0.454
**Injured Body Part**			
Multiple	--	--	0.454
Head/face	0.944	0.055-16.327	0.969
Extremities	1.855	0.208-16.501	0.58
Abdomen/thorax	0	0	1
Spine	11.333	0.765-167.971	0.078
Other Regions	2.833	0.152-52.738	0.485
**Disability**			
**Injury Category**			
Primary	1.874	0.531-6.612	0.329

Concussion	--	--	1
Blast Lung	0	0	1
Tympanic Membrane Rupture	0	0	1
Secondary	1.146	0.440-2.981	0.78
Traumatic Amputation	--	--	0.652
Concussion	2.625	0.22-31.349	0.446
Lacerations	0.946	0.095-9.378	0.962
Penetrating Injury	1.667	0.257-10.792	0.592
Tertiary	0.812	0.331-1.991	0.649
Concussion	--	--	0.455
Blunt Injury	0.412	0.09-1.881	0.252
Crush Syndrome	1.167	0.094-14.518	0.905
Quaternary	0	0	0.999
Environmental Contamination	--	--	1
Burns	0	0	0.999
Toxic Gas Inhalation	0	0	1

Multiple Categories	1.891	0.473-7.564	0.368
**Injured Body Part**			
Multiple	**--**	**--**	0.301
Head/face	0.464	0.072-2.976	0.418
Extremities	0.236	0.052-1.072	0.062
Abdomen/thorax	0	0	1
Spine	2.167	0.262-17.892	0.473
Other Regions	0	0	0.999

* Significance (p-value ≤0.05)

† Abbreviations: OR=Odds Ratio; CI= Confidence Interval
